# Effects of Cooling on Ankle Muscle Strength, Electromyography, and Gait Ground Reaction Forces

**DOI:** 10.1155/2014/520124

**Published:** 2014-05-04

**Authors:** Amitava Halder, Chuansi Gao, Michael Miller

**Affiliations:** ^1^Division of Ergonomics and Aerosol Technology, Department of Design Sciences, Faculty of Engineering, Lund University, P.O. Box 118, 221 00 Lund, Sweden; ^2^Division of Physiotherapy, Department of Health Science, Faculty of Medicine, Lund University, P.O. Box 157, 221 00 Lund, Sweden

## Abstract

The effects of cooling on neuromuscular function and performance during gait are not fully examined. The purpose of this study was to investigate the effects of local cooling for 20 min in cold water at 10°C in a climate chamber also at 10°C on maximal isometric force and electromyographic (EMG) activity of the lower leg muscles. Gait ground reaction forces (GRFs) were also assessed. Sixteen healthy university students participated in the within subject design experimental study. Isometric forces of the tibialis anterior (TA) and the gastrocnemius medialis (GM) were measured using a handheld dynamometer and the EMG was recorded using surface electrodes. Ground reaction forces during gait and the required coefficient of friction (RCOF) were recorded using a force plate. There was a significantly reduced isometric maximum force in the TA muscle (*P* < 0.001) after cooling. The mean EMG amplitude of GM muscle was increased after cooling (*P* < 0.003), indicating that fatigue was induced. We found no significant changes in the gait GRFs and RCOF on dry and level surface. These findings may indicate that local moderate cooling 20 min of 10°C cold water, may influence maximal muscle performance without affecting activities at sub-maximal effort.

## 1. Introduction

Temperature is considered to be significant determinant of skeletal muscle function and performance [1, 2]. Muscle force generation and power output vary with changes in body temperature. An optimal temperature range at which the best performance of muscle isometric contraction occurs has been described [3, 4]. It has also been demonstrated that muscle contraction forces and rate of force development were impaired at low muscle temperatures [5, 6] and voluntary muscular force production capabilities were reported to be affected below 27°C without core temperature change [2]. In fact, Rutkove showed that even a low degree of cooling decreased the rate of muscular force production [7].

Various peripheral cooling protocols have been used to explore the effects on muscle performance. De Ruiter et al. [1] investigated the effect of temperature on the rate of isometric force development in the lower arm following 20 min immersion in water baths at 37°, 31°, 25°, and 22°C and found that isometric force was reduced below 25°C. Cold water immersion of a limb causes a higher rate of heat loss compared to other environmental cooling. There are various sports and recreational activities both in water and land, where exposure to cold environments may affect power generation [3]. A recent study by Cè et al. [8] showed that both fatigue and muscle cooling decreased nerve conduction velocity. Local vasoconstriction in tissues exposed to cold is likely to decrease oxygen extraction and impair the oxidative reactions [9]. It has also been reported that muscle fatigue due to high intensity dynamic work developed earlier when the muscle temperature was decreased [10].

Several experiments that have been performed in humans and in rats show that muscle shortening velocity and isometric muscle tension were altered by decreasing temperature [11]. Drinkwater and Behm found that the rate of isometric twitch and tension force development declined by 50% and 46%, respectively, when the plantar flexors were cooled to 22°C [12]. Davies et al. [13] reported that local cooling to 24°C of the triceps muscle reduced its maximal voluntary contraction (MVC). Oksa et al. [14] found reduced maximal muscle performance during dynamic exercise performed at a lowered room air temperature (10°–5°C) and Ranatunga et al. [15] summarized that maximum voluntary tension remained relatively stable on cooling to 25°C but varied by about 30% on cooling to 12°–15°C. However, Meigal et al. [16] reported that exposure to cold room air (10°C) for 30 min did not impair the MVC during isometric elbow flexion.

Studies have also shown that ankle dorsiflexion (DF) and plantar flexion (PF) strengths are strongly correlated to walking speed [17]. During gait the reciprocal activity of agonist and antagonist muscles is important for the production of optimal force and for coordination [18]. Oksa et al. [19] argued that muscle activation changes, due to cooling and rewarming, are centrally regulated due to muscle agonist/antagonist pattern changes followed by cooling. As muscle function is affected by cooling, one may assume that postural balance and gait performance may also be affected. Local leg cooling techniques using water immersion have been used to induce some degree of foot sole anesthesia and the effects on postural control have been examined. Eils et al. [20] and Stål et al. [21] used ice water as a cooling method to assess the effects on muscle function during gait by using data from force plates and electromyography. However, few studies have investigated electromyographic activity and ground reaction forces specifically during gait following local muscle cooling by water immersion. Petrofsky and Lind [22], Bigland-Ritchie et al. [23], and De Luca [24] investigated the effect of cooling on EMG frequency during isometric and submaximal exercise. The results showed increased amplitude of surface EMG but decreased center frequency power spectra with lower muscle temperature and also revealed evidence of muscle fatigue through cooling the muscles in water from 40° to 10°C [22].

Biomechanics of gait can be affected due to cooling of lower extremity muscles [20]. As a result, one may have poor coordination or may even slip and fall. The required coefficient of friction (RCOF) has been used in several studies to ascertain the minimum friction required at the foot or shoe and floor interface as an important biomechanical parameter for evaluating slips and falls [25]. To the best of our knowledge, the effects of lower leg muscle cooling on both EMG and RCOF have not been well documented. Neither has ankle muscle strength, ground reaction forces during gait, and EMG activity following lower leg muscle cooling been adequately investigated. Therefore, further exploration of muscle performance in cold environments is required [2].

The objectives of this study were to assess the effects of lower leg cooling on maximum ankle DF and PF muscle forces, EMG, GRF, and RCOF during gait in healthy young subjects. It was primarily hypothesized that, as a consequence of cooling the muscles of the lower leg, maximum isometric force would be reduced and changes may occur in the muscle EMG recordings. It was hypothesized primarily that consequences of cooling would reduce maximum isometric force and affect muscle electrical activities of the lower extremities. The secondary hypothesis was that local cooling would modify the gait strategies in anterior-posterior and vertical components of the ground reaction forces, the required coefficient of friction, and the electromyographic properties during walking.

## 2. Materials and Methods

### 2.1. Participants

Sixteen university students participated in the within subject design study, 8 males and 8 females. Physical and anthropometric characteristics of the participants were age, 27.0 ± 2.9 years; body mass, 66.3 ± 9.8 kg; height, 169.5 ± 7.8 cm, and body mass index (BMI) 23.0 ± 2.5. All volunteer participants signed an informed consent form. All participants were clinically healthy, no neurological or musculoskeletal abnormalities and no previous history of lower limb injuries which might influence normal walking and balance ability. Subjects were requested to abstain from alcoholic beverages and intensive physical exercise 24 hours prior testing. A complete explanation of the purpose of the study and experimental procedures was described and the participants were also informed of the risk of experiencing some discomfort. The study was approved by the regional ethical review board in Lund (EPN) and performed according to declaration of Helsinki for research involving human subjects.

### 2.2. Experimental Design

A repeated measure of the same subject design was used with two independent variables by using two experimental stations. One of them was in a “room temperature environment” (air temperature was about 21°C) where the walkway with force plate was installed. The other experimental station was the “cold climate chamber” at 9.5°C, which is 0.5°C lower than target water temperature 10°C with a chair and two 60 liter plastic water baths in front of the chair in order to immerse both lower legs while sitting. The purpose of the cold chamber was to maintain cold water temperature at 10°C during the 20 min immersion period [1, 20, 26]. A physical examination bench was also placed inside the climate chamber to accommodate lying position for the dynamometer measures. Skin temperatures were measured using an infrared thermometer (Agema Infrared Systems, model-TPT64P, Germany).

### 2.3. Experiment Protocol

See Figure 1.

### 2.4. Cooling and Temperature Measurements

#### 2.4.1. Before Cooling

EMG electrodes were attached to the muscle belly of the GM muscle and the TA muscle. After-wards, pre cooling (Tsk-pre) skin temperature was measured on GM and TA muscle bellies next to the EMG electrodes. Isometric ankle DF and PF dynamometer testing was performed on dominant leg in the supine position with the knee straight. Gait over the force plate and EMG measurements were performed immediately after the dynamometer testing.

#### 2.4.2. Muscle Cooling

**Figure 2:**
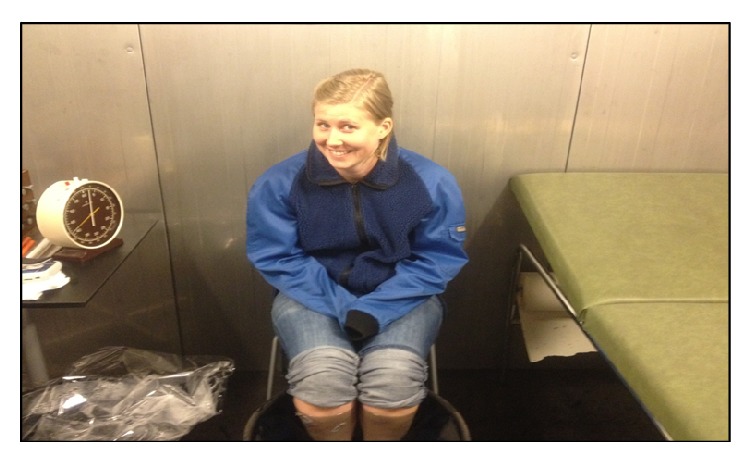
Subject's both legs were immersed in a cold water bath in the cold climate chamber.

Water-resistant adhesive tape was applied over the EMG electrodes to prevent water influx into electrodes. In the cold chamber, the subjects were dressed in light winter jackets with the trousers folded up above both knees, and gloves and thin woolly caps were provided upon individual demands. The cold water volume was approximately four times more than leg volume to maintain a temperature of 9.5 to 10.5°C (Figure 2).

#### 2.4.3. After Cooling

Again skin temperature (Tsk-post 1) was obtained from the same locations that were dried quickly with a towel; then the isometric DF and PF tests were performed. Immediately after the isometric tests, the Tsk-post 2 was recorded with the subjects still being in the 9.5°C cold chamber. The postcooling walking trials were then performed at ambient room temperature on the force plate walkway in the laboratory. Finally, the Tsk-post 3 was taken at room temperature after the gait trials.

### 2.5. Instrumentation

#### 2.5.1. Dynamometer

Maximum isometric voluntary force measurements were performed using the Lafayette Handheld Dynamometer, model LA-01163, IN, US.

**Figure 3:**
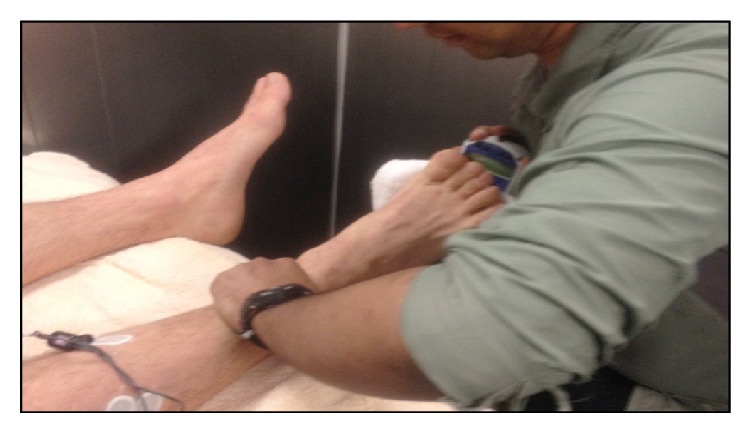
Measuring plantar flexion force using the hand held dynamometer.

*Dynamometer Procedure*. All the dynamometer PF and DF, MVC tests were carried out by the same assessor. In the supine position, the subjects were instructed to fixate themselves by firmly gripping the sides of the bench and maintain the hips and knees extended. The assessor stabilized the lower leg by manual fixation proximal to the ankle joint to minimize substitution movements [17] (Figure 3). To measure DF force, the dynamometer was applied against the dorsal surface of the foot just proximal to the metatarsal heads. For PF measurement, the dynamometer was applied to the sole of the foot just proximal to the metatarsal heads. An extra soft towel was placed between the dynamometers contact plate and the subjects' feet for comfort. To standardize the positions for the isometric tests, PF testing was performed at maximal subtalar end range of DF and the DF tests were performed at comfortable subtalar end range of PF. The ankle joint (talocrural) was positioned at midrange of motion in each direction. Before the force measurements the subjects practiced the two movements in order to avoid confusion and minimize the training effect during the actual dynamometer tests. Three consecutive MVCs of the ankle DF and PF were registered using the “make” technique, where the examiner held the dynamometer stationary while the subject exerts a maximal force against it [17, 27]. Subjects were asked to build their force to maximum as hard as possible up to two-second period. By increasing force gradually in this manner it was easier for the tester to hold the dynamometer stationary against the subject's exertion. Subjects thereafter continue at a maximum effort for another 4-5 seconds until the “beep” sound from the dynamometer. A brief pause allowed for about 30 seconds of recovery between contractions to avoid fatigue. Strong verbal encouragement was given during each contraction. If the difference between scores was within 3 kilograms, the test was considered complete; otherwise, the test was repeated.

#### 2.5.2. Walkway and Force Plate

**Figure 4:**
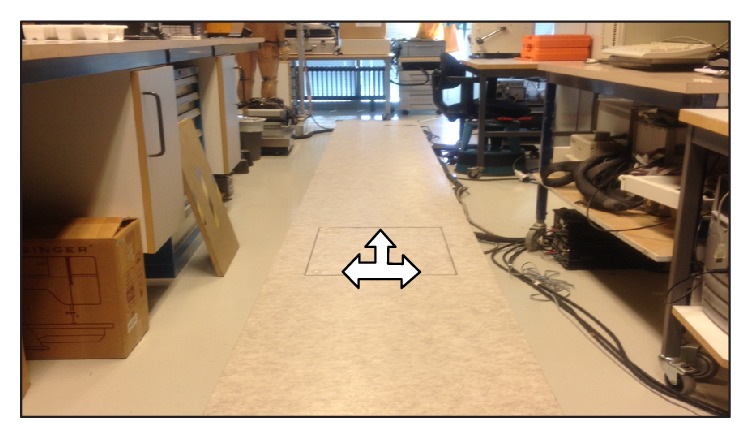
Force plate installed in laboratory walkway.

*Walkway*. The laboratory wooden walkway with a vinyl surface was of the following dimensions, 0.6 m wide and 7.2 m long (Figure 4).

*Force Plate*. A force plate Kistler 9281B (Switzerland) (Figure 4) was incorporated into the walkway. The DAQ System 5695A1 and Amplifier type 9865A with BioWare Software (version 4.1.0.2, type 2812A-04) were used for data collection. The force plate was placed in the walkway at 4.1 m and was also layered with vinyl sheet and lay flush with the walkway.

*Gait Protocol*. Each subject was instructed to walk barefooted at a comfortable habitual pace and not to focus on the force plate. Each subject was first given the opportunity to become familiar with the walkway by walking across the force plate so that the dominant foot struck the force plate area.

#### 2.5.3. EMG

The Megawin (ME6000-T16 Mega Electronics, Kuopio, Finland) System Biomonitor and Software “Megawin” version 3.1-b10 were used to record and analyze surface EMG data. The sampling rate was set at 1024 Hz. The skin was prepared by shaving if necessary then lightly abraded with fine sand paper and cleaned with isopropyl alcohol to minimize impedance. The pregelled bipolar surface electrodes (Ambu Neuroline 720) were placed adjoining in the fiber direction at the upper third portion of the GM and TA muscles. The ground electrodes were fixed over noncontractile tissue. Water-proof adhesive tape was also applied. The same investigator attached all electrodes using the SENIAM recommendations.

### 2.6. Data Processing and Analysis

#### 2.6.1. Strength Measurements

The means of the three maximum voluntary of contractions (MVCs) for each muscle pre- and postcooling were calculated and used in the statistical analysis.

#### 2.6.2. EMG

The raw EMG signal was processed in the following way: the DC offset using the inbuilt Megawin software was applied. The raw EMG signal was digitally band pass filtered (20–400 Hz); a notch filter 50 Hz was also applied to clean the signal from eventual artifacts from the surrounding electrical appliances: the signal was then full-wave rectified and root mean square (RMS) averaging was applied. From the processed signals the peak and median values in *μ*V were obtained. The mean of the three highest EMG peaks for each MVC trial for each muscle pre-and postcooling was calculated. Markers on the EMG recording were used to identify the period of onset and end of each gait trial and the median EMG for each period was recorded. The mean values of the medians pre- and postcooling were used in the analysis. The peak EMG values for each muscle and subject were used to normalize the EMG values from the gait tests [28].

#### 2.6.3. Ground Reaction Forces

Three forces from the force plate, namely, vertical force (*F*_*z*_), lateral shear force (*F*_*x*_), and anterior-posterior shear force (*F*_*y*_), were obtained using 8 channels. The GRF data were processed using a zero lag low-pass Butterworth 4th order filter with a cut-off frequency of 100 Hz and then normalized to each subject's body weight. Time was normalized to 100% of the stance phase with 0% being heel strike (HS) and 100% representing toe-off (TO) [29, 30]. The body weight was weighed on the force plate in order to normalize the ground reaction forces (N) to the subjects' respective body weights (N).

The stance period was identified as follows: onset began when *F*_*z*_ exceeded 10 N and the end of the stance phase was determined when the vertical force dropped below 10 N. The force plate stance phase period was divided into two percentage subphases (HS and TO) from the midpoint of stance phase using the time slice options in the “Bioware”. The HS and TO phases were associated with the actions of the DF and PF. The average of HS and TO peaks from the three trials was calculated for each subject and the mean values of all sixteen subjects were compared for the pre- and postcooling conditions.

#### 2.6.4. Required Coefficient of Friction (RCOF)

**Figure 5:**
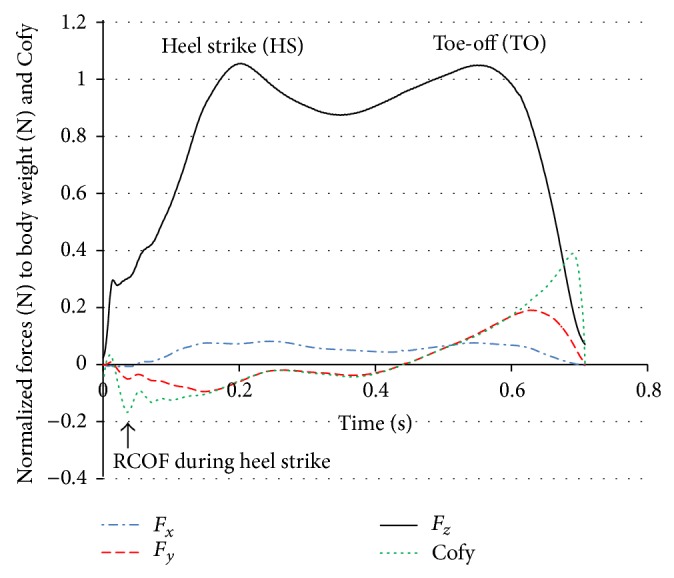
Example of GRFs and RCOF used for analysis, the vertical force (*F*_*z*_), longitudinal shear force (*F*_*y*_), and transverse force (*F*_*x*_) during the HS and TO phases. The second peak of Cofy indicated by an arrow at the bottom represents the RCOF during heel strike.

The ratio of shear to normal ground reaction force, termed the required coefficient of friction (RCOF) [29], was calculated for each trial by dividing longitudinal anterior-posterior force by vertical force (*F*_*y*_/*F*_*z*_). According to Chang et al. [25] RCOF was generally accepted to be the value of the third peak of the RCOF versus time curve, as shown in (Figure 5) below. The third peak was scrutinized from GRF curve by selecting the time period from 20 milliseconds to 200 milliseconds after heel contact (when *F*_*z*_ reached 10 N); however, on a few occasions the third peak was unclear so the second peak was used instead.

### 2.7. Statistical Analysis

Paired two-tailed *t*-tests were used to test for differences between pre- and postcooling for the maximal strength measurements, mean EMG from the maximal strength measurements, the median EMG during gait trials, and the mean of the peak GRFs and the RCOF. When the normality was challenged for the EMG data, Wilcoxon Signed-Rank Test was also performed. However, the statistical results were in essence similar to the *t*-test results (Table 1). Significance was set at *P* ≤ 0.05 throughout and exact *P* values are given above 0.05. All data were processed and analyzed using SPSS v.20, IBM Corp., US.

## 3. Results

### 3.1. Cooling of Lower Legs

**Figure 6:**
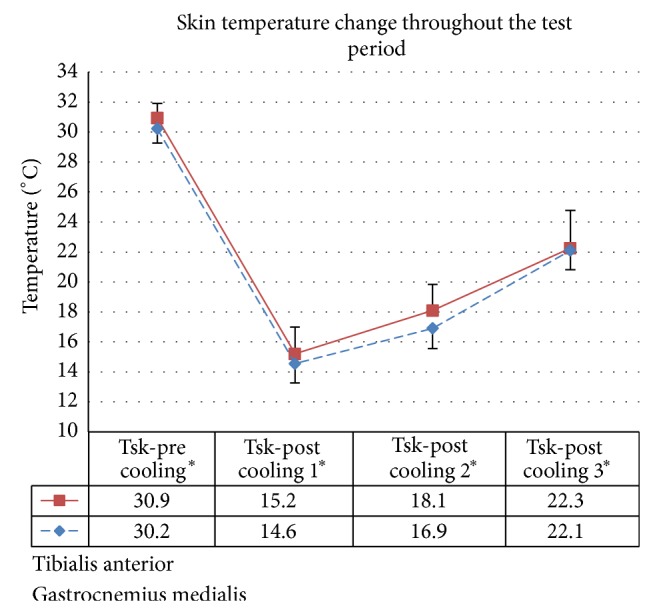
Mean skin temperature over the two muscles (TA and GM) for four measurement phases. Tsk-pre: before cooling, Tsk-post cooling 1: immediately after cooling, Tsk-post cooling 2: after the postcooling MVC test, and Tsk-post cooling 3:end of gait trials.

All sixteen subjects successfully completed 20 minutes of cooling on both legs in cold water at 10°C in the cold chamber. The average skin temperatures over the two muscles (TA and GM) decreased by about 15.7°C and 15.6°C, respectively, after 20 minutes of cooling. Most of the subjects reported reduced sensation after 20 minutes cooling at 10°C, which were gradually regained during walking trials. Skin temperatures gradually increased by about 2.9 and 2.2°C for TA and GM, respectively, after postcooling MVC tests, even though the tests were performed in the cold chamber. At the end of all gait trials, skin temperatures increased by about 4 to 5°C. The skin temperature changes over the two muscles throughout the test period are shown in Figure 6.

### 3.2. The Effects of Cooling on Strength MVC

There was a significantly reduced isometric maximum force in TA muscle (*P* < 0.001) after cooling, and the GM muscle force difference was not significant (Table 1).

### 3.3. The Effects of Cooling on EMG during MVC

The mean EMG amplitude of GM muscle was significantly increased by approximately 400 *μ*V after cooling (*P* < 0.003). There was no difference for the TA muscles (Table 1).

### 3.4. The Effects of Cooling on the EMG of Gait Muscles

The average of the median for the three gait trials after cooling was significantly higher in the GM (*P* < 0.001) compared to the precooling value. There was no difference for the TA muscles (Table 1).

### 3.5. The Effects of Cooling on Gait Ground Reaction Forces

We found no differences in the GRFs between pre- and postcooling. Neither did the third peak of coefficient of friction result in a significant difference (*P* = 0.851) (Table 2).

## 4. Discussion

The present study investigated the effects of cooling on ankle muscle maximum isometric force, ground reaction forces, and EMG activity of the TA and GM during MVC trials and gait trials. The main findings showed that cooling decreased maximum force of the TA but not the GM muscle. The study also showed that cooling increased the EMG amplitude significantly of the GM but not the TA muscle during the maximum voluntary contractions and gait trials.

### 4.1. The Cooling Effect on Ankle Muscle Strength

A significant decrease in MVC strength in the order of 10% was recorded in the TA after cooling; however, there was no change for the GM. Several studies have shown that cooling with water decreases isometric strength in superficial muscles [1, 13]. Holewijn and Heus [5] also monitored a drop in maximal handgrip force after 30 min local cooling at 15°C. Comeau et al. [31] evaluated the effects of environmental cooling on force production in the thigh muscles and registered a significant force drop in the quadriceps and hamstrings at temperatures 10°C or below. We have only found in the present study that the TA significantly decreased in MVC performance. One possible explanation for this discrepancy may be the morphological differences of the muscles. The TA muscle has a much lesser volume than the GM muscle; however, the muscle fiber length is almost the double. The internal cooling of the muscles could not be measured in this study and cooling may have been greater in the TA and thus nerve conduction and the contraction process at sarcomere level may be more affected [2, 6, 7, 23]. Furthermore, the common peroneal nerve that innervates the TA is very superficial and therefore quite exposed to the cold water which may result in lower conductibility [8, 32] and thereby possible lesser force production in accordance with findings by Hsu and Stevenson [33]. Moreover, the TA has a muscle fiber length twice the length of GM, which allows for a more favorable force generation compared with the plantar flexors [34]. One other factor is that the deep soleus muscle also contributes to plantar flexor strength and it is quite possible that the soleus was less affected by the cooling resulting in a less affected force production. Despite the fact that surface skin temperature did decrease significantly, more invasive techniques are required to register the core muscle temperature. Another possible criticism may be the limitations of using a handheld dynamometer; however, the hand held dynamometer has been demonstrated reasonably reliable and valid to measure the foot and ankle strength of young and old adults [17, 35, 36].

### 4.2. The Cooling Effect on EMG Values

We found a significant and large increase in the mean EMG amplitude for the GM muscle during the MVC trials after cooling and we also found similar results in the normalized median EMG during the gait trials (Table 1). It is known that EMG amplitude of the fatigued and cooled muscles increases on isometric and submaximal exercise due to altered motor unit recruitment and activation [22, 23, 37]. It is possible that the cooling effect in this experiment induced an EMG fatigue response of the GM. Previous studies have revealed that PF was more prone to fatigue after a prolonged level running; however DF is less subjected to fatigue during a prolonged five-hour hilly running than that of PF studied by Fourchet et al. [38]. Taken together, for the TA we found a lower maximal force production immediately after cooling but no difference in the mean EMG, and for the GM we found no difference in maximal force production after cooling but a very large and significant increase in the mean EMG amplitude.

### 4.3. Muscle Cooling and Ground Reaction Forces during Gait

The analysis of ground reaction forces showed no significant alterations on peak vertical and longitudinal ground reaction forces. Other studies have shown that cooling of the feet to numbness with ice water close to 0°C did produce force changes such as, a delay in the force distributions during heel strike [20, 21]. In our study the cooling temperature was 10°C; thus cutaneous sensation on the soles of the feet may not have been functionally impaired. Hreljac reported that the DF muscles perform at or near maximum capacity when a transition of speed is required and this may make the muscle susceptible to overexertion [39]. Neptune et al. [40] argued that the muscle TA is primarily responsible for helping to toe-off in early swing, while the plantar flexors support the body during propulsion and at swing initiation in late stance.

RCOF is the ratio of *F*_*y*_ over *F*_*z*_ and is used to assess slip risk. If available coefficient of friction in GRF is smaller than that of RCOF, a slip will occur. Reductions in the relative magnitude of the shear forces were more than the vertical forces resulting in the overall effect of a decrease in the peak RCOF [29]. Again, we found no difference in RCOF values between pre- and postcooling which may also be explained by the low gait speed in a secure environment. Since the average peak vertical ground reaction force was not significantly changed, the time component was primarily responsible for the nonsignificant decrease in vertical impulse. In other words, either the extra time necessary to produce force following cooling or the muscle contraction time is slower [6, 41]. One probable explanation to the nonsignificant results for the force plate values is that the gait task used in this study was not demanding enough. Gait speed, although not measured, was relatively slow in the confines of the laboratory with a short 3.5 m approach to the force plate and moreover, the slip risk on dry vinyl floor surface was relatively small.

Cold water exposure has been demonstrated to increase joint stiffness due to increased viscosity of the joints' synovial fluid [42–44]. Increased joint stiffness may also alter postural control and muscle force production. However, some researchers have found that the muscle cooling itself has no effect on force production and that a decrease in nerve conduction velocity is a primary contributor to the decrease in isometric maximal performances [45–47].

To increase our knowledge of EMG, muscle force, and ground reaction force changes, further studies need to be performed with more extreme cooling and at more vigorous and prolonged duration of muscle intensity and repetitive activities. Mean power frequency and the ratio between MVC and EMG (MVC/EMG) of the TA and GM could be further analyzed. In application to injury management, the results from this study imply that the treatment of injuries by cooling may affect muscular performance immediately after treatment. The results also indicate that neuromuscular control and/or muscular homeostasis regulation may be negatively affected by the degree of cooling described in this laboratory study and may have implications for motor performance during cold exposure in recreational, sports, and occupational activities. The findings may assist researchers in evaluating physical performance after cold exposure. The cooling procedure described may be applied in studies of sportsmen and workers who need to perform in wet and cold conditions.

## 5. Conclusion

Cooling of both lower legs in cold water at 10°C and in a climate chamber with an air temperature of 10°C for 20 minutes resulted in a decrease of the maximal isometric voluntary force in the TA muscles but not in the GM muscles in young healthy individuals. The mean EMG amplitude during the maximal force tests was unchanged in the TA muscle but significantly increased in the GM perhaps indicating an induced fatigue response. Force plate reaction forces and RCOF during gait were not affected on a dry and level walkway. In conclusion, 20 min cooling in cold water at 10°C can influence our maximum muscle performance, but the cooling may not be severe enough to have some impact on daily submaximal activities.

## Figures and Tables

**Figure 1:**
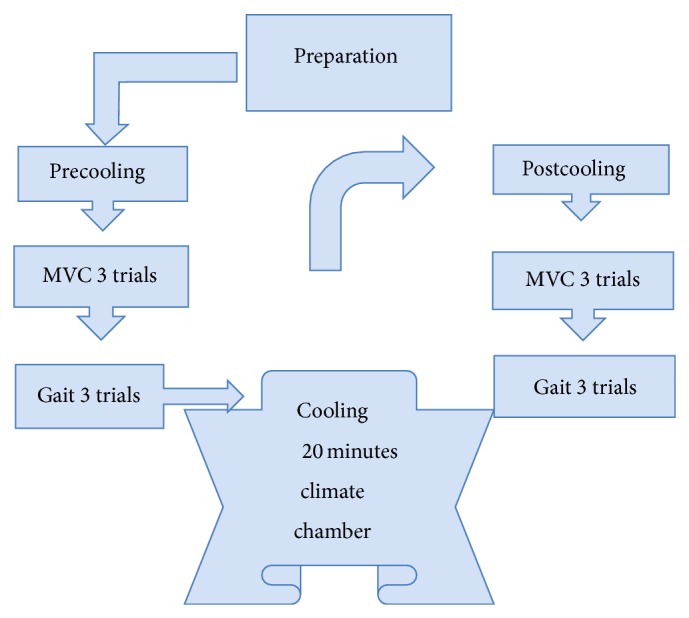
The procedure of experimental study: each subject performed 3 maximal voluntary contractions (MVCs) and 3 gait trails during the pre- and postcooling stages. The subjects performed precooling trials, followed by 20 min of cold water immersion at 10°C, followed by the trials immediately after cooling.

**Table 1:** The means and standard deviations (mean ± SD) of the three MVC trials as well as the mean EMG amplitude during the three MVC trials and normalized mean of the three median EMG values from the three gait trials are given for the TA and GM muscles before and immediately after cooling (*n* = 16).

	Dorsiflexion (TA muscle)			Plantar flexion (GM muscle)		
	Precooling	Postcooling	*t*-test	Precooling	Postcooling	*t*-test
MVC (Kg)	23.8 ± 2.7	21.3 ± 2.7	<0.001	30.6 ± 4.5	29.7 ± 3.0	0.221
EMG during MVC (µV)	446.4 ± 218.2	443.4 ± 250.7	0.939	284.8 ± 128.3	705.5 ± 487.7	0.003
Normalized EMG in gait trials (%)	11.3 ± 5.3	13.1 ± 7.3	0.158	13.6 ± 10.3	19.8 ± 11.9	<0.001

**Table 2:** Vertical and longitudinal GRFs (normalized by body weight in Newton) (mean ± SD) during heel strike and toe-off in relation to DF and PF, respectively, and RCOF before and after cooling (*n* = 16).

	Heel strike (HS) phase			Toe-off (TO) phase		
	Precooling	Postcooling	*t*-test	Precooling	Postcooling	*t*-test
Peak vertical (*F*_*z*_)	1.14 ± 0.11	1.17 ± 0.10	0.140	1.15 ± 0.08	1.14 ± 0.06	0.186
Peak longitudinal (*F*_*y*_)	0.21 ± 0.05	0.21 ± 0.06	0.903	0.24 ± 0.03	0.23 ± 0.03	0.129
Required coefficient of friction (RCOF)∗	0.26 ± 0.04	0.26 ± 0.05	0.851			

^*^RCOF during heel strike phase was included in the analysis.
